# Maternal Weight and Gestational Diabetes Impacts on Child Health: A Narrative Review

**DOI:** 10.7759/cureus.70192

**Published:** 2024-09-25

**Authors:** Rahul Khandelwal, Revat J Meshram, Ankita Patel, Sneha Reddy, Yogesh B Manek

**Affiliations:** 1 Pediatrics, Jawaharlal Nehru Medical College, Datta Meghe Institute of Higher Education and Research, Wardha, IND; 2 General Surgery, Jawaharlal Nehru Medical College, Datta Meghe Institute of Higher Education and Research, Wardha, IND

**Keywords:** birth weight, child health outcomes, gestational diabetes, long-term metabolic risk, maternal obesity, pregnancy complications

## Abstract

Maternal weight and gestational diabetes mellitus (GDM) have significant implications for both maternal and child health. This narrative review explores the intricate relationship between maternal pre-pregnancy weight, gestational weight gain, and GDM, focusing on their short- and long-term effects on child health outcomes. The review highlights evidence linking maternal obesity and GDM to increased risks of adverse outcomes in infants and children, such as macrosomia, neonatal hypoglycemia, childhood obesity, and metabolic disorders. It also discusses the intergenerational transmission of metabolic risk, underscoring the importance of early intervention and prevention strategies in maternal healthcare. Furthermore, the review emphasizes the need for tailored approaches to managing maternal weight and GDM to mitigate potential risks and improve health outcomes for future generations. Key recommendations include promoting healthy preconception weight, monitoring gestational weight gain, and enhancing postnatal follow-up for both mothers and children. This review underscores the critical role of maternal health in shaping the developmental trajectory of offspring and highlights opportunities for healthcare professionals to reduce the long-term impact of GDM on child health.

## Introduction and background

Maternal health plays a crucial role in shaping the mother's and her offspring's well-being. Among the significant maternal health issues, maternal obesity and gestational diabetes mellitus (GDM) have emerged as critical public health concerns. Globally, the prevalence of maternal obesity has been on the rise, with studies reporting that nearly one in five pregnant women are obese [1]. This rise in obesity rates has paralleled an increase in the incidence of GDM, a condition that affects approximately 7%-10% of pregnancies [2]. Maternal obesity and GDM have been linked to a range of adverse outcomes, both for the mother and the developing child. The impact of maternal weight and gestational diabetes extends beyond pregnancy, significantly influencing the health trajectory of the child. Maternal obesity is associated with increased risks of fetal macrosomia, neonatal complications, and long-term childhood obesity [3]. Additionally, children born to mothers with GDM have a higher likelihood of developing metabolic disorders, such as type 2 diabetes and cardiovascular diseases, later in life [4]. This intergenerational transmission of health risks underscores the critical need to address maternal weight and GDM not only for maternal health but also for improving child health outcomes.

This narrative review aims to examine the impacts of maternal weight and gestational diabetes on child health. Specifically, it will explore the mechanisms by which maternal obesity and GDM affect fetal development and early childhood health, review the short- and long-term health outcomes for children, and highlight potential interventions to mitigate these risks. The review seeks to inform healthcare strategies and policies that can improve maternal and child health outcomes by synthesizing current evidence.

## Review

Search methodology

The search methodology for the narrative review involved a comprehensive and systematic approach to identifying relevant literature. The review utilized multiple electronic databases, including PubMed, Scopus, and Google Scholar, to gather peer-reviewed articles, clinical studies, and review papers published between 2000 and 2024. Search terms included “maternal weight,” “gestational diabetes,” “child health,” and related keywords, combined using Boolean operators to refine results. Inclusion criteria focused on studies examining the direct and indirect effects of maternal weight and gestational diabetes on child health outcomes. Articles were screened based on relevance, quality, and methodological rigor. Data extraction was performed to compile findings on how maternal weight and gestational diabetes influence child health, including growth, development, and long-term health consequences.

Maternal weight and its impact on child health

Overview of Maternal Weight Categories

Maternal weight during pregnancy can be categorized into four main groups: underweight, average weight, overweight, and obesity. These categories are determined by pre-pregnancy Body Mass Index (BMI). According to the World Health Organization (WHO), the BMI cutoffs are as follows: underweight (BMI <18.5), average weight (BMI 18.5-24.9), overweight (BMI 25-29.9), and obesity (BMI ≥30). Maternal weight plays a significant role in both maternal and child health outcomes. Each weight category has distinct risks and impacts on birth outcomes, childhood development, and long-term health [5].

Impact on Birth Outcomes

Maternal weight significantly influences birth outcomes, with both underweight and overweight categories being associated with adverse consequences. Underweight mothers are at a higher risk of preterm birth and low birth weight (LBW) infants [6]. Preterm birth, defined as birth before 37 weeks of gestation, is a major cause of neonatal morbidity and mortality [7]. On the other hand, maternal overweight and obesity are associated with an increased risk of large-for-gestational-age (LGA) infants and macrosomia, defined as a birth weight greater than 4,000 grams [8]. These infants are more likely to require interventions such as cesarean delivery due to their size and may experience complications like shoulder dystocia.

Impact on Childhood Development

The influence of maternal weight extends beyond birth, affecting childhood cognitive and behavioral development. Research has shown that maternal obesity is linked to cognitive outcomes and increased risk of neurodevelopmental disorders, such as poorer attention-deficit/hyperactivity disorder (ADHD) [9]. Overweight and obesity during pregnancy may lead to higher risks of emotional and behavioral difficulties in children. Additionally, maternal undernutrition, common in underweight mothers, can impair fetal brain development, potentially resulting in long-term cognitive deficits [10].

Long-Term Health Consequences for the Child

**Figure 1:**
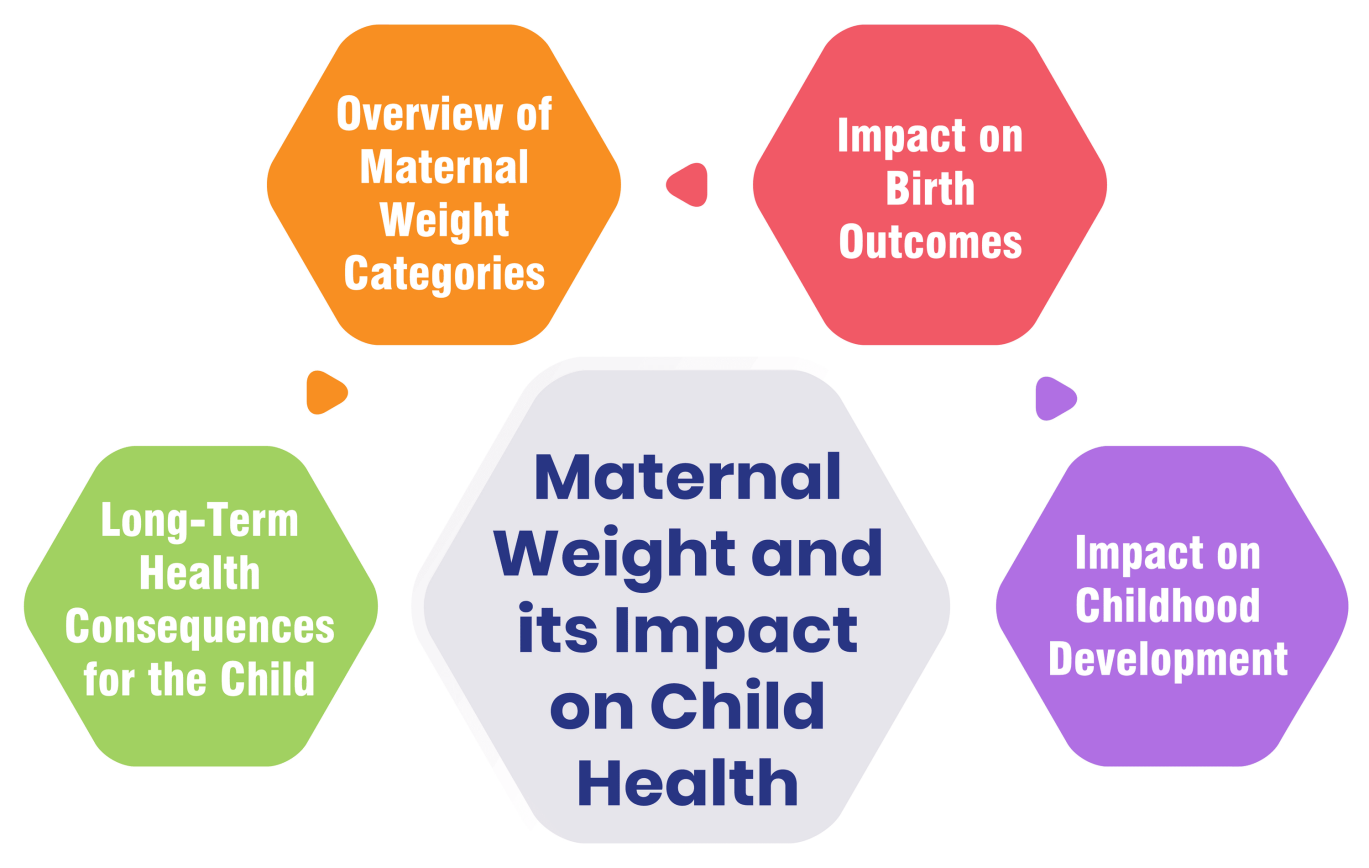
Maternal weight and its impact on child health Image Credits: Dr. Rahul Khandelwal

The child's long-term health is significantly influenced by maternal weight during pregnancy. Children born to mothers with obesity have a higher likelihood of developing childhood obesity themselves, perpetuating a cycle of weight-related health issues. This increased risk of obesity also predisposes children to metabolic syndrome, which includes a range of conditions such as insulin resistance, hypertension, and dyslipidemia [11]. Additionally, maternal overweight and obesity have been associated with an elevated risk of cardiovascular diseases in offspring later in life due to early-life programming of the cardiovascular system [12]. In contrast, children of underweight mothers may face growth retardation and developmental delays, further highlighting the importance of optimal maternal weight. Figure 1 shows maternal weight and its impact on child health.

GDM and its impact on child health

Definition and Prevalence of GDM

GDM is a form of glucose intolerance during pregnancy. It is typically diagnosed in the second or third trimester and resolves after delivery. The pathophysiology of GDM involves insulin resistance that exceeds the body's compensatory insulin secretion, leading to hyperglycemia. Globally, GDM affects 5%-15% of pregnancies, though the prevalence varies based on the population studied and the diagnostic criteria used [13]. The International Association of Diabetes and Pregnancy Study Groups (IADPSG) and the World Health Organization (WHO) have established diagnostic criteria for GDM, focusing on fasting and postprandial blood glucose thresholds [14].

Impact of GDM on Neonatal Health

The effects of GDM on neonatal health are significant and include several short-term complications. One of the most common issues is neonatal hypoglycemia, which occurs due to increased fetal insulin production in response to maternal hyperglycemia [15]. Respiratory Distress Syndrome (RDS) is also more likely in infants born to mothers with GDM, potentially due to delayed fetal lung maturity [16]. In addition, GDM is associated with an increased risk of neonatal jaundice, a condition linked to higher bilirubin levels in newborns [17].

Long-Term Health Consequences for the Child

Children born to mothers with GDM are at an increased risk of developing long-term health problems. Obesity in childhood and adolescence is one of the most well-documented outcomes [4]. This is attributed to in-utero exposure to hyperglycemia, which affects fetal adiposity. Moreover, these children have a higher risk of developing type 2 diabetes and insulin resistance later in life [3]. Research suggests that the intrauterine environment, altered by maternal hyperglycemia, may program the child’s metabolic pathways, increasing susceptibility to metabolic disorders [18].

Impact on Child’s Metabolic Health

**Figure 2:**
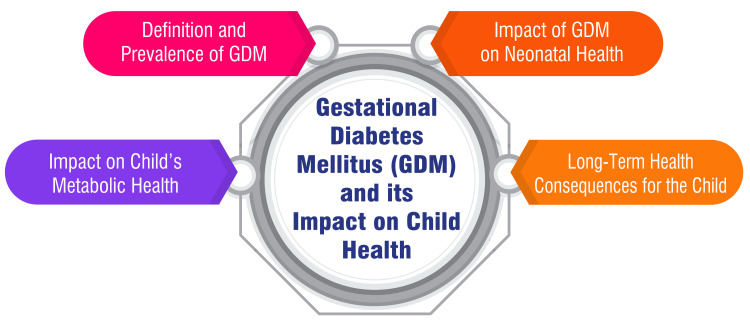
Gestational diabetes mellitus (GDM) and its impact on child health Image Credits: Dr. Rahul Khandelwal

Maternal GDM can also affect the child's glucose metabolism, contributing to an increased likelihood of metabolic syndrome in later life [19]. Studies indicate that the offspring of mothers with GDM have altered insulin sensitivity, placing them at greater risk for metabolic disorders like hypertension and dyslipidemia [20]. Figure 2 shows GDM and its impact on child health.

Interaction between maternal weight and GDM on child health

Combined Impact of Maternal Obesity and Gestational Diabetes

Maternal obesity and GDM are both independent risk factors for adverse health outcomes in offspring. Still, when combined, they exhibit synergistic effects on the child’s long-term health. Studies have demonstrated that children born to mothers with both obesity and GDM are at an increased risk of developing obesity themselves, as well as metabolic disorders such as type 2 diabetes, cardiovascular diseases, and insulin resistance [21,22]. Furthermore, these children often show altered early-life growth patterns, such as rapid weight gain during infancy, which is a predictor of childhood obesity [23]. This synergistic impact may be due to excessive nutrient transfer to the fetus and abnormal metabolic environments during pregnancy, contributing to long-term health consequences.

Potential Genetic and Epigenetic Mechanisms

The interaction between maternal weight and GDM can influence fetal development through genetic and epigenetic mechanisms. Fetal programming, influenced by maternal metabolic conditions, predisposes offspring to metabolic diseases later in life [4]. GDM and maternal obesity can lead to changes in gene expression and modifications in epigenetic marks, such as DNA methylation and histone modifications. These changes can affect genes involved in glucose metabolism, insulin sensitivity, and adipogenesis [24]. For example, studies have shown that maternal hyperglycemia and obesity alter fetal insulin-like growth factor (IGF) pathways, promoting the development of adiposity and metabolic syndrome in children [25].

Environmental and Lifestyle Factors

The postnatal environment, including diet and physical activity, also plays a significant role in modulating the health outcomes of children born to mothers with obesity and GDM. Although genetic and epigenetic mechanisms establish a predisposition to metabolic disorders, environmental factors can either exacerbate or mitigate these risks [26]. For instance, children exposed to unhealthy postnatal environments characterized by poor diet, sedentary behavior, and limited physical activity are more likely to develop obesity and related metabolic diseases. Conversely, a healthy postnatal environment with proper nutrition and regular physical activity can help counterbalance the risks posed by maternal obesity and GDM [27]. Furthermore, breastfeeding has been found to offer protective effects against the development of obesity and metabolic disorders in children exposed to maternal GDM and obesity during pregnancy [28].

Preventive measures and interventions

Maternal Weight Management During Pregnancy

Managing maternal weight during pregnancy is crucial for both maternal and fetal health. The Institute of Medicine (IOM) provides guidelines for appropriate weight gain based on pre-pregnancy BMI. For instance, underweight women are advised to gain between 28-40 pounds, while women with obesity should aim for a gain of 11-20 pounds [29]. Healthy lifestyle practices, including balanced nutrition and regular physical activity, are essential to avoid excessive weight gain, associated with increased risks of GDM, hypertension, and LGA infants [30]. Proper weight management during pregnancy has been shown to reduce these risks and promote better maternal and infant health outcomes.

Screening and Management of Gestational Diabetes

Early detection and management of gestational diabetes is vital to minimizing its impacts on both maternal and child health. Screening for GDM typically occurs between 24 and 28 weeks of gestation, although women with higher risk factors may be screened earlier [31]. Management of GDM includes dietary modifications, regular blood glucose monitoring, and, in some cases, insulin therapy. A low glycemic index diet and regular physical activity are recommended to maintain glycemic control [6]. These interventions help reduce the risk of complications such as macrosomia, preeclampsia, and neonatal hypoglycemia.

Impact of Interventions on Child Health

Prenatal interventions for weight management and GDM have a profound impact on child health outcomes. Effective management of maternal weight gain and glycemic control can reduce the risk of neonatal complications such as preterm birth, RDS, and type 2 diabetes later in life [4]. Postnatal interventions, including breastfeeding and continued healthy maternal nutrition, further enhance infant growth and development. Research indicates that maternal lifestyle modifications during pregnancy benefit immediate neonatal outcomes and positively influence long-term child metabolic health [32].

Role of Healthcare Providers in Prevention and Education

**Figure 3:**
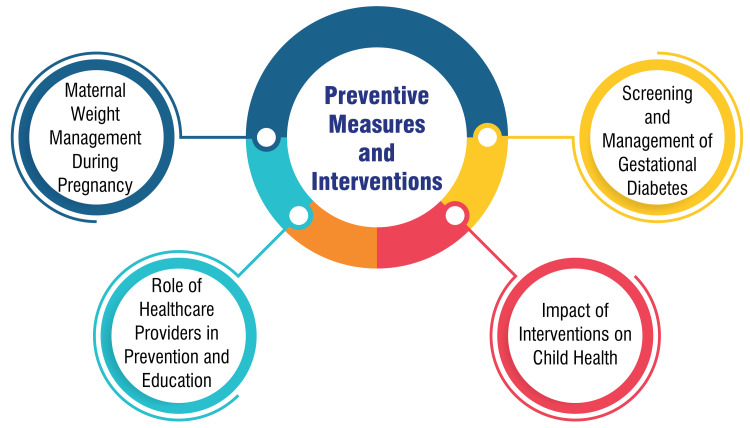
Preventive measures and interventions Image credits: Dr. Rahul Khandelwal

Healthcare providers play a critical role in preventing adverse outcomes related to maternal weight and gestational diabetes. Providers are responsible for educating pregnant women about the importance of weight management, proper nutrition, and regular physical activity. They also guide women through the screening process for GDM and develop personalized care plans to manage the condition [2]. Healthcare professionals, including obstetricians, midwives, and dietitians, are key resources in promoting health literacy and facilitating preventive measures that benefit both mother and child. Figure 3 shows preventive measures and interventions.

Challenges and limitations in research

Heterogeneity in Study Designs

One of the significant challenges in understanding the impacts of maternal weight and gestational diabetes (GDM) on child health is the heterogeneity in study designs. Research in this area often utilizes varied methodologies, ranging from observational studies to randomized controlled trials (RCTs), each with differing measures for maternal weight, GDM diagnosis, and child health outcomes. This lack of standardization can make it difficult to compare results across studies and draw robust conclusions. For example, a systematic review by Nehring et al. highlighted inconsistencies in how maternal pre-pregnancy BMI and child obesity outcomes were measured across studies, complicating the synthesis of findings [33].

Limitations of Current Data

There are notable gaps in the longitudinal data necessary to fully understand the long-term effects of maternal weight and gestational diabetes on child health. Many studies do not follow children into adolescence or adulthood, which limits the understanding of how early exposures to maternal obesity or GDM might influence health outcomes later in life, such as metabolic syndrome or cardiovascular disease. Additionally, certain populations, such as minority groups and low-income families, are often underrepresented in research, which limits the generalizability of the findings. Confounding factors, such as socioeconomic status, breastfeeding, and lifestyle habits, can also complicate the interpretation of results [3].

Ethical Considerations in Maternal Health Research

Ethical concerns are another limitation in researching maternal weight and gestational diabetes, particularly when pregnant women and children are involved. Research involving pregnant women requires careful ethical consideration due to the potential risks to both the mother and fetus. Additionally, conducting interventions or clinical trials can be challenging because of the ethical obligations to avoid harm to either party. Studies must balance the need for robust data collection with the duty to protect vulnerable populations [34].

Future directions

Future research on the impacts of maternal weight and gestational diabetes on child health should prioritize longitudinal studies to assess the long-term outcomes for children born to mothers with these conditions. Investigating the effectiveness of early intervention strategies in mitigating adverse health effects on offspring, including personalized nutrition and lifestyle modifications, is crucial [35]. Additionally, exploring the role of epigenetic mechanisms and maternal metabolic profiles in shaping child health outcomes can provide insights into preventative measures [36]. Integrating advanced data analytics and machine learning approaches to predict and personalize care plans could enhance early detection and management strategies [37]. Collaborative efforts between researchers, healthcare providers, and policymakers are essential to translate findings into actionable guidelines for improving maternal and child health [38].

## Conclusions

In conclusion, this narrative review underscores the significant impact of maternal weight and gestational diabetes on child health, highlighting a complex interplay of factors that influence long-term outcomes. Maternal obesity and gestational diabetes not only elevate the risk of adverse birth outcomes but also set the stage for a range of chronic conditions in offspring, including metabolic syndrome and obesity. The review emphasizes the need for early intervention and comprehensive management strategies to mitigate these risks, including preconception counselling, optimal weight management, and effective glucose control during pregnancy. Addressing these issues through targeted public health initiatives and personalized care approaches is essential for improving maternal and child health and reducing the burden of these intergenerational health challenges.
